# Artificial Intelligence-Based Cyber-Physical System for Severity Classification of Chikungunya Disease

**DOI:** 10.1109/JTEHM.2022.3171078

**Published:** 2022-04-28

**Authors:** Dilbag Singh, Manjit Kaur, Vijay Kumar, Mohamed Yaseen Jabarulla, Heung-No Lee

**Affiliations:** School of Electrical Engineering and Computer ScienceGwangju Institute of Science and Technology 65419 Gwangju 61005 South Korea; Department of Computer Science and EngineeringNIT Hamirpur 219797 Hamirpur 177005 India

**Keywords:** Artificial intelligence, cyber-physical system, automated diagnosis, Chikungunya disease, random forest, adaptive crossover, genetic algorithm, severity classification

## Abstract

Background: Artificial intelligence techniques are widely used in solving medical problems. Recently, researchers have used various deep learning techniques for the severity classification of Chikungunya disease. But these techniques suffer from overfitting and hyper-parameters tuning problems. Methods: In this paper, an artificial intelligence-based cyber-physical system (CPS) is proposed for the severity classification of Chikungunya disease. In CPS system, the physical components are integrated with computational algorithms to provide better results. Random forest (RF) is used to design the severity classification model for Chikungunya disease. However, RF suffers from overfitting and poor computational speed problems due to complex architectures and large amounts of connection weights. Therefore, an evolving RF model is proposed using the adaptive crossover-based genetic algorithm (ACGA). Results: ACGA can efficiently optimize the architecture of RF to achieve better results with better computational speed. Extensive experiments are performed by utilizing the Chikungunya disease dataset. Conclusion: Performance analysis demonstrates that ACGA-RF achieves higher performance as compared to the competitive models in terms of F-measure, accuracy, sensitivity, and specificity. The proposed CPS system can prevent users from visiting hospitals and can render services to patients living far away from hospitals.

***Clinical translation statement:*** The proposed model can be utilized for the severity classification of Chikungunya disease. The research findings are impactful as the proposed model can prevent users from visiting hospitals and can render services to patients living far away from hospitals.

## I. Introduction

The Healthcare system plays a vital role in the development of any nation. Government is responsible to design proper healthcare policies to protect their citizens from any outbreak of diseases [1]. Hence, the outbreak of any new disease such as coronavirus is a major challenge for the healthcare system. Several viruses exist that can affect both animals and human beings. Chikungunya is one of the viruses that can be spread very rapidly and may create a big problem for the health system. Two basic types of infected mosquitoes i.e., Aedes albopictis and Aedes agypti transmit this virus in the human body [2]. The symptoms of Chikungunya are joint pain, sudden high fever, and rash. Some infected persons have headaches, fatigue, digestive complaints, and conjunctivitis [3]. The symptoms of Chikungunya are very similar to dengue because the same mosquito carries both viruses [4]. But in Chikungunya, joint pain is more severe as well as redness of the eyes. The symptoms of sore throat are different from dengue infection. Chikungunya may not cause death. As per literature, it is found that the patient recovers within a week of this disease [5]. But, the joint pain may last for a few months. The doctors start the treatment by perceiving the symptoms of patients. However, the exact measurement of these symptoms is not possible. Therefore, the treatment of patients may not be effective.

Instead of symptoms found in the patients, reverse transcription-polymerase chain reaction (RT-PCR) and serological tests are used to diagnose Chikungunya. Both tests require blood samples of patients [6]. However, these tests are unable to provide reliable performance for this disease. Supervised learning techniques such as machine learning and deep learning can be used to evaluate the severity of this disease by considering the symptoms of patients and laboratory tests [7]. The severity classification of Chikungunya infected persons is still an ill-posed problem.

In [8], a fog-based framework for Chikungunya disease diagnosis was designed. J48 was utilized to classify Chikungunya infected patients. In [9], wearable internet of things (IoT) and fog-based framework for classification and controlling the Chikungunya disease was proposed. Fuzzy-C means (FCM) classifier was utilized for Chikungunya classification. But J48 [8] and FCM [9] suffer from over-fitting and hyper-parameters tuning problems. In [10], a particle swarm optimization-based ANFIS (PANFIS) model was implemented for the diagnosis of Chikungunya disease. Initially, an adaptive neuro-fuzzy inference system (ANFIS) classifier was used to classify the infected patients. Thereafter, particle swarm optimization (PSO) was utilized to overcome the parameter tuning problem with ANFIS. It achieved remarkable results compared to artificial neural networks (ANN). But, PANFIS [10], [11] suffers from the over-fitting problem. Also, sometimes PSO may be stuck in local optima and suffers from premature convergence problems [12], [13].

Therefore, to overcome the over-fitting and hyper-parameters tuning problems, an efficient evolving Random forest (RF) model is proposed for the severity classification of Chikungunya disease. The main contributions of this paper are as follows:

1) A cyber-physical system (CPS) based severity classification model is proposed for Chikungunya disease. In CPS system, the physical components are integrated with computational algorithms to attain better results.
2) Evolving RF model is proposed for severity classification of Chikungunya disease. An adaptive crossover-based genetic algorithm (ACGA) is utilized to evolve RF model.
3) Deep learning model is also implemented and compared with ACGA-RF for severity classification of Chikungunya disease.

The remainder of this paper is organized as follows. Section II presents the related work. The proposed ACGA-RF model is described in Section III. Performance analyses are presented in Section IV. The concluding remarks are discussed in Section V.

## II. Related Work

Artificial intelligence techniques are widely used in solving medical problems. Recently, researchers used machine learning techniques for the classification of Chikungunya disease. Hossain *et al.* [5] utilized the different symptoms of patients for the accurate assessment of Chikungunya disease. Their proposed framework collected the data from the interviews of patients. They used a belief-based rule system for predicting the level of Chikungunya. Their model attained an accuracy of 92%. Yang [14] developed a decision system for the diagnosis of Chikungunya disease. The neural network was used for classification by considering the uncertainty of the disease’s symptoms. However, the uncertainty of some symptoms is not considered in this approach. Ganesan *et al.* [15] presented three different models to diagnose the Chikungunya disease. However, these models require human intervention for the assessment of this disease. Caicedo-Torres *et al.* [16] proposed a machine learning-based classifier for differentiating the dengue and Chikungunya patients. Their classifier was tested on 447 patients. The logistic regression model outperformed the other models. The accuracy obtained from logistic regression was 87%.

Ibrahim *et al.* [17] presented the backpropagation method for predicting the epidemic disease. They used epidemic disease factors for prediction. Thereafter, these factors were applied to the clustering technique. Their method is capable to identify the epidemic disease using feature classification. Coelho *et al.* [18] used a transfer learning model for predicting mosquito-borne diseases. They used time-series data from two Brazilian cities. Both the long short-term memory neural network model and random quantile forest model provided the same prediction performance. Caicedo-Torres *et al.* [19] utilized the machine learning techniques for envisaging the morbidity of Chikungunya in Colombia. Kernel ridge regression was used for forecasting the Chikungunya cases. Cross-validation and mean absolute error were used.

Sippy *et al.* [20] developed two prediction models on the Machala dataset. The first model namely the severity index for suspected arbovirus model (SISA) that utilized demographic data. Another model namely the severity index for suspected arbovirus (SISA1) with laboratory utilized the laboratory data. The accuracies obtained from SISA and SISAl were 91% and 95%, respectively. Both models are capable to envisage arbovirus hospitalization.

Shimpi *et al.* [21] used a backpropagation algorithm to predict the Chikungunya disease. Five gradient-based optimization techniques were used. The pre-processed features were applied to the backpropagation algorithm for classification. The accuracy obtained from this model was 95%. However, a small dataset was used for validation purposes. Eng *et al.* [22] used machine learning techniques for predicting the binding affinity of T-cell epitopes of Chikungunya. They built prediction models for identifying binders and non-binder. This model will be helpful for vaccine development.

From the existing literature, it is found that the majority of the existing models suffer from hyper-parameters tuning, (i.e., optimization of initial control parameters), over-fitting and data insensitivity problems [23], [24]. Hence, there is a need to develop an efficient model for severity classification for Chikungunya disease.

## III. Proposed Framework

**FIGURE 1.**
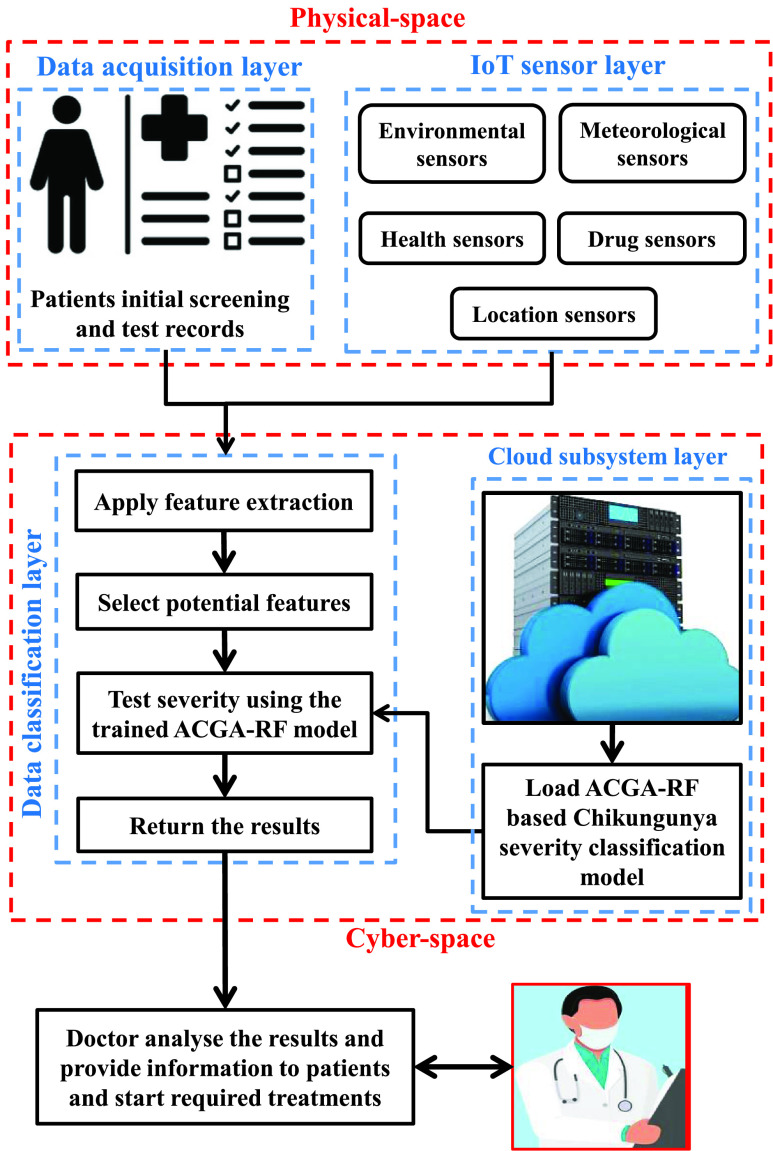
Proposed ACGA-RF model based Chikungunya severity classification model.

Motivated from [8], [10], an efficient model is proposed in this paper for diagnosis of Chikungunya disease. Figure 1 shows the proposed artificial intelligence-based cyber-physical system (CPS) for the diagnosis of Chikungunya disease. There are two main components in the proposed model, i.e., physical space and cyberspace. In physical space. the data related to users’ health is collected and forwarded to cyberspace for predicting the severity of the Chikungunya virus [25], [26]. In cyberspace, there are two sub-layers, i.e., the cloud subsystem layer and data classification layer [27], [28]. In the cloud subsystem layer, data and the proposed trained model are stored. At the data classification layer, the severity prediction of the Chikungunya virus is done in real-time by applying the proposed ACGA-RF model after extracting the potential features. Finally, doctors are involved for further assessment of the results.

### A. Physical Space

In physical space, there is a data acquisition layer that collects users’ personal data, symptoms related to Chikungunya, and records of initial screening with the help of sensors.

The Chikungunya dataset is obtained from [8] and [9]. Sood and Mahajan [8] and [9] built the dataset by taking the symptoms-based dataset from [29] that contains eleven health features, i.e., abdominal pain, muscle pain, bleeding disorder, fatigue, eyes pain, itching, nausea, sore throat, joint pain, skin rash, and fever, of 2367 patients. Dataset with 5032 cases comprising environmental variables was taken from [30] and [31]. Monthly climate variables, i.e., rainfall, temperature, and humidity, were taken from [32]–[34]. The symptoms-based data was combined with climate and environmental features to validate ACGA-RF (for further details refer to [8] and [9]).

Table 1 shows the common features of Chikungunya virus. It contains several sensing devices to observe the health of patients. The observed information is then transferred to the data classification layer to classify the health of a particular client. The description of the dataset is mentioned in Table 1 (refer [8], [10]).

1) User Personal Data: This comprises the user’s personal data. GPS sensors are used to collect data such as UID, name, age, address, and mobile number.
2) Health-related Data: This set consists of vital signs. It provides information related to rashes on the skin, muscle pain, appetite loss, joint pain, fever, redness in the eyes, headache, nausea, sore throat, fatigue, and vomiting. Biosensors are used to collect this information.
3) Environmental and location Information: It provides the position of patients, susceptible users, uninfected users, and positions of mosquito dense areas, water quality, humidity, and mosquito breeding sites. These positions are evaluated by Global Positioning System (GPS) sensors to obtain the travel history of every patient. Radio Frequency Identification (RFID) tags and mosquito sensors are also utilized to store the proximity interactions between infected/uninfected/ susceptible users, mosquito densities, and breeding sites.

**TABLE 1:** Data Attributes Related to Chikungunya Virus

Attribute type	Attribute Name	Sensors used
User Personal data	UID (unique identification), Name, Age, Gender, Location of user workplace, Location of user residence, and Mobile number	GPS sensors
Health-related data	Joint pain, Muscle Pain, Rashes on the skin, Appetite loss, Fever, Redness in the eyes, Headache, nausea, sore throat, fatigue, vomiting	Biosensors
Environmental and location data	Water quality, air temperature, humidity, mosquito density, rainfall, mosquito breeding sites, level of carbon dioxide	Mosquito and climatic sensors

Table 2 depicts the personal-parameters of registered users. It presents a brief description of the user’s personal parameters such as name, age, gender, address, mobile number, and contact details of guardians (refer [8], [10]). Table 3 shows Chikungunya symptoms such as headache, exposure to the risky area, nausea, fever, rash, vomiting, etc.

**TABLE 2:** Chikungunya’s User Personal-Parameters

Sr. No.	Parameters	Explanation
1.	Name	Patient name
2.	Age	Age of user in years
3.	Gender	Male or Female
4.	GPS	Geographic location of user
5.	Address	Address of the registered patient
6.	Telephone No.	Mobile number
7.	Patient Guardian’s	Contact details of guardians

**TABLE 3:** Chikungunya Symptoms Related-Dataset

Sr. No.	Attributes	Decision	Description
1	Headache	Binary	Whether a user has a headache
2	Exposure to risky area	Binary	Whether a user works or lives in risk area
3	Nausea	Binary	User is feeling tired and sleepy
4	Fatigue	Binary	User feels weak and loss weight
5	Rash and Vomiting	Binary	Itching problem and has body marks
6	Muscular Pain	Binary	User suffers from muscular pain
7	Joints Swelling	Binary	User suffers from swelling
8	Joint pain	Binary	User suffers from joint pain symptom
9	Fever	Integer	Temperature in °C
10	Initiation post infection	Yes	3 to 7 days

### B. Cyber Space

Cyberspace is comprised of two sub-layers, i.e., the cloud subsystem layer and the data classification layer. The detail of registered users and the proposed trained model is stored in cloud subsystem layers. Whereas, at the data classification layer, diagnosis of the Chikungunya virus is done by applying ACGA-RF to the potential features. Also, RF-based model is used as it has the abilities of generalization, learning, fault tolerance, and adoption. However, RF model is sensitive to its control parameters. An efficient tuning of these control parameters can improve the performance of RF. Therefore, to automatically optimize control parameters of RF, an ACGA-RF model is proposed. Finally, the predicted severity report of a patient is transmitted to doctors for further treatment.

## IV. Proposed Evolving Random Forest Model

This section discusses the proposed evolving random forest model. Initially, RF model is briefly discussed to understand its basic notations and hyper-parameters. Random forest (RF) is an ensemble of classification/regression trees [35], where every tree shows a mapping from feature space to the response. Trees can be obtained either using a subsampled data set of actual data or bootstrapped. Every tree is conditionally independent of one another. However, RF model is sensitive to its architecture and hyper-parameters. Thus, an adaptive crossover genetic algorithm (ACGA) is utilized to obtain the optimized architecture and hyper- parameters of RF.

In literature, genetic algorithm is widely accepted to optimize various classifiers such as deep learning models [23], [36]. Genetic algorithms utilize crossover and mutation operators during evolution phases to obtain the final solution. It has been found that the selection of efficient crossover and mutation operators is a challenging problem. To efficiently select crossover operator(s), Xue *et al.* [37] proposed an adaptive crossover genetic algorithm (ACGA). A group of crossover operators was utilized during the evolution process. Based on the performance of crossover operators, roulette wheel selection was utilized to select a specific crossover. In this paper, ACGA [37] is utilized to form evolving RF model. Three different crossover operators are used. The working of the proposed ACGA-RF is depicted in Algorithm 1.

Algorithm 1 **ACGA Based RF**
Input: Max population ( $M$), $A_{S}$, iterative threshold ( $IT$), No. of crossovers ( ${C}$), and No. of fitness evaluations ( ${FE}$)
Output: Optimized values for RF
1: P $\leftarrow $ init_P(M)
2: Initialize $rW$, $pL$, ${nR}_{IT \times {C}}$, and ${nP}_{IT \times {C}}$.
3:
$\widehat {P} = {p_{1}, p_{2}, \ldots, p_{C} } \leftarrow init\_{}A({C})$
4:
$nFE \;\leftarrow \; 0$ and $k \;\leftarrow \; 0$
5:
$P_\delta \leftarrow \phi $
6: **while** $nFE < {FE}$ **do**
7: **for** $i = 1 \; \text {to}\; M/2$ **do**
8:
$a_{v} \leftarrow $init_R ( $\widehat {P}$)
9: Two offsprings are selected as parents: $O_{p}$
10:
$O_{c} \leftarrow $init_C( $O_{p}$, $a_{v}$)
11:
$O_{c} \leftarrow $init_M( $O_{c}$)
12:
$nFE \; \leftarrow \; nFE + 2$
13:
$[rW, pLw]~\leftarrow $ init_CA( $O_{p}$, $O_{c}$)
14: Add $O_{c}$ to $P_\delta $
15: **end for**
16: k $\leftarrow $ k +1
17: Update $rW$ to $k^{th}$ row of ${nR}_{IT \times {C}}$
18: Update $pL$ to $k^{th}$ row of ${nP}_{IT \times {C}}$
19: **if** k = IT **then**
20:
$\widehat {P} \leftarrow $init_A( ${nR}_{IT \times {C}}$, ${nP}_{IT \times {C}}$)
21:
$k = 0$
22: **end if**
23:
$R \leftarrow P \cup P_\delta $
24:
$P \leftarrow $init_S( $R$)
25: Assign non-dominated offsprings in $P$ to $T_{P}$
26: Optimized values $\leftarrow ~T_{P}$
27: **end while**
28: **return** Optimized values

**init_P():** Population ${P}$ is created using normal distribution by creating $M$ vectors. Each vector represents the architecture and hyper-parameters-related values of RF.

**init_A():** Adaptive crossover selector ( $A_{S}$) is utilized by assigning probabilities to each crossover as $1/{C}$. ${C}$ shows a number of utilized crossovers.

**init_R():** Given crossover is selected according to roulette wheel selection and probabilities obtained from $A_{S}$.

**init_C():** Apply selected crossover on parents’ offspring to form child offsprings.

**init_M():** Mutation operator is utilized to obtain child offspring. Compute child offsprings are saved in the offspring population( $P_\delta $).

**init_CA():** Dominated offsprings are then evaluated by using the actual and child offsprings. The respective outcomes are saved in $rW$ and $pL$. $P_\delta $ is computed during ${M/2 }^{th}$ step.

**init_S():** Crowded distance [38] and Non-dominated sorting [39] are used to obtain $M$ solutions from $R$ ( $P~\cup ~P_\delta $).

**init_D():** To allocate reward/ penalty to selected offsprings, dominance comparison is utilized.

Penalty and reward of offsprings is saved in ${nP}_{IT \times {C}}$ and ${nR}_{IT \times {C}}$, respectively. After $IT$ number of phases, $A_{S}$ is updated by considering ${nP}_{IT \times {C}}$ and ${nR}_{IT \times {C}}$. All steps are repeated until the termination criterion (i.e., $FE$) is satisfied.

The succeeding subsections discuss the steps of ACGA.

### A. Fitness Function

Fitness function is designed to optimize RF by using sensitivity and specificity. It is defined as:

\begin{equation*} \max F(X)=\{ f_{1} (X)~\; \text {and} \; f_{2}(X) \}\tag{1}\end{equation*}

Here, $X$ is an offspring. $f_{1}$ and $f_{2}$ represent the sensitivity and specificity parameters, respectively.

### B. Crossover Operators

Three different crossover operators, i.e., single-point [38], chaotic crossover [40], and reduced surrogate [41], [42] are utilized. Single-point [38] has significant results to solve many computationally hard problems. It has shown better computational speed compared to the existing crossover operators [37].

Reduced surrogate [41], [42] can avoid unnecessary crossover operations when parents have similar offspring. Initially, it evaluates parents and forms a group of crossover points where both parents have different genes. In the absence of such a crossover point, no crossover operator is implemented. Chaotic crossover [40] can obtain a better converged and distributed group of Pareto-optimal offspring.

### C. Penalty and Reward

Penalties and rewards are allocated to offsprings by utilizing two matrices namely $pL$ and $rW$ as:

\begin{align*} rW=&{[0 \;.\;.\;. 0]}_{1\times {C}} \tag{2}\\ pL=&{[0 \;.\;.\;. 0]}_{1\times {C}}\tag{3}\end{align*}

Pareto optima among the offsprings is used to modify $rW$ and $pL$.

#### 1) Parent is Non-Dominated

Pareto optima is evaluated between child and respective parent offsprings. If child offsprings are dominated by parents, then append $pL_{q}$ by 1, otherwise append $rW_{q}$ by 1. The pseudocode of updation of penalty and reward is depicted in Algorithm 2.

Algorithm 2 **Credit Allocation (init_CA ())**
Input: Parents ( $\rho $), Children ( $\delta $), crossover selected using init_A ( $q$)
Output:
$rW$, $pL$ [ $n_{d}$, $d_{s}$] $\leftarrow $ init_D( $\rho $) // $d_{s}$ and $n_{d}$ define dominated and non-dominated offsprings, respectively.
1: Dominated parent (assume $\rho _{1} \prec \rho _{2}$).
2: **if** $d_{s} \neq \phi $ **then**
3: **for** i = 1 to 2 **do**
4: **if** $\rho _{1} \prec \delta _{i}$ **then**
5:
$pL_{q} \leftarrow \,\,pL_{q} + 1$
6: **else**
7:
$rW _{q} \leftarrow rW rW_{q} + 1$
8: **end if**
9: **end for**
10: **else**
11: // If parent is non-dominated.
12: **for** i = 1 to 2 **do**
13: **if** $\rho _{1} \nprec \delta _{i}~\rho _{2} \nprec \delta _{i}$ **then**
14:
$rW _{q} \leftarrow rW rW_{q} + 1$
15: **else**
16:
$pL_{q} \leftarrow pL_{q} + 1$
17: **end if**
18: **end for**
19: **end if**
20: P $\leftarrow $ init_P (M)
21: **return** Optimized values for RF

#### 2) Dominated Parent

If parent $1~(\rho _{1})$ is dominated by parent 2 ( $\rho _{2}$), then pareto optima of each child offspring is compared with $\rho _{2}$. If child offspring is dominated by $\rho _{2}$, then append $pL_{q}$ by 1. Otherwise, update $rW_{q}$ by 1.

### D. Updation of Adaptive Crossover Selector

$A_{S}$is utilized to update the crossover selection probabilities. During evolution process, it is implemented after every $IT$ steps (refer [43]). Two matrices, i.e., ${nP}_{IT\times {C}}$ and ${nR}_{IT\times {C}}$ are used to hold the values of $pL$ and $rW$, respectively. Recently updated $IT$’s $pL$ and $n_{R}$ values are utilized to modify $A_{S}$. To evaluate the probability for $q^{th}\; (q = 1, 2,\ldots, {C})$ crossover, addition of $q^{th}$ column is utilized (refer [43]).

## V. Performance Analysis

To analyze the efficiency of proposed model, health-related attributes are collected from [8], [34], [44], [45]. It mainly consists of attributes such as age, sex, location, fever, skin rashes, and joint pain. It is collected for approximately 10000 users. Around 1805 users are at no risk while 3890 are at normal risk and 4305 are at high risk. The acquired data is stored in cloud storage and is used for recognition by an optimized RF model. ACGA-RF is compared with J48, SVM, ANN, RF, adaptive neuro-fuzzy inference system (ANFIS), PANFIS [10], and deep learning (DL).

To implement DL model, various layers (i.e., feature input layer, fully connected layer, batch normalization layer [46], [47], ReLU layer [46], [47], softmax layer [46], [47], and classification layer) are utilized. For normalizing the input data, Z-score normalization [48] is used. Minibatch size is set to be 8. Adam [49] a stochastic gradient descent optimizer is used to achieve the better convergence of DL model.

### A. Training Analysis

**FIGURE 2.**
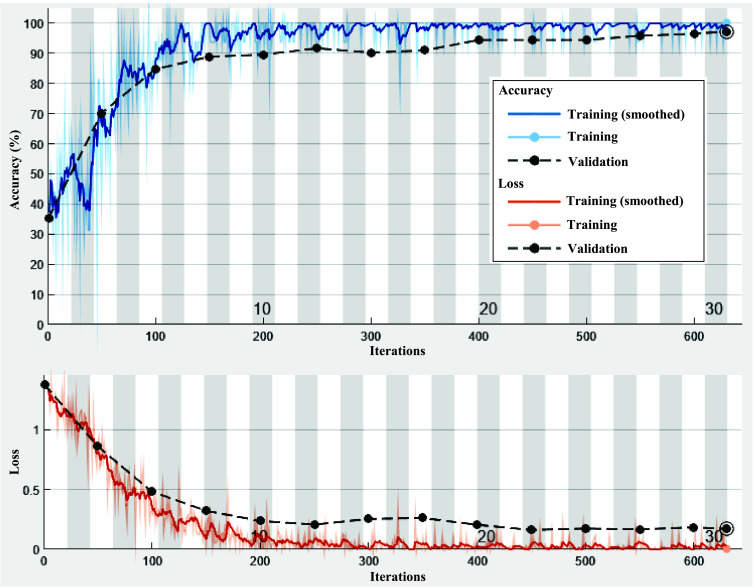
Accuracy and loss analysis of the deep learning-based severity classification of Chikungunya disease.

Figure 2 shows the accuracy and loss analysis of the deep learning-based severity classification model for Chikungunya disease. It clearly shows that the deep learning achieves significantly better convergence speed. It achieves 98.3% validation accuracy, therefore, least affected by the impact of over-fitting as training accuracy is 100%.

The performance of RF achieves the best training and validation accuracy values are 99.5% and 97.6%, respectively. Although it shows remarkable results, the performance of the proposed approach is still far from the optimal results. It shows an over-fitting problem as there is a high difference between training and validation accuracy values. The performance of ACGA-RF model achieves the best training and validation accuracy values are 100.0% and 99.6%, respectively. Thus, ACGA-RF model achieves remarkable results than both deep learning and RF models. Also, ACGA-RF is least affected by the over-fitting issue as there is only a 0.4% difference between the training and validation accuracy which are 1.7 and 1.9 for deep learning and RF, respectively. Also, ACGA-RF shows an enhancement in validation accuracy over deep learning and RF as 1.3% and 2.0%, respectively.

### B. Confusion Matrix Analysis

To evaluate the performance of ACGA-RF, confusion matrix analyses are also achieved. Table 4 depicts the confusion matrix analysis proposed ACGA-RF, RF, and deep learning (DL) models. It is found that RF achieves the testing accuracy of 97.54%. It is found that the deep learning (DL) model achieves the testing accuracy of 98.31%. DL shows an average improvement of 0.77% over RF model. It is found that ACGA-RF achieves an accuracy of 99.43%. Thus, the proposed ACGA-RF achieved an average improvement of 1.89% and 1.12% over RF and deep learning-based severity classification models, respectively.

**TABLE 4:** Confusion Matrix Analysis Among the Proposed ACGA-RF, DL, GA-RF, PSO-RF, and RF Models

Model	RF	DL	GA-RF	PSO-RF	ACGA-RF
TP	203	206	207	207	209
TN	314	315	315	317	318
FP	7	4	3	3	1
FN	6	5	5	3	2
Accuracy	0.9754	0.9831	0.9837	0.9855	0.9943
Sensitivity	0.9712	0.9763	0.9732	0.9855	0.9905
Specificity	0.9781	0.9874	0.9690	0.9902	0.9968
F-measure	0.9747	0.9818	0.9829	0.9902	0.9936

### C. Comparative Analysis

Boxplot and ANOVA are used for statistical analysis. The hypotheses for every performance measure can be defined as:

\begin{align*} \begin{cases} H_{0} & \mu M_{1} = \mu M_{2} = \ldots. = \mu M_{7},\\ H_{A} & \text {Means are not equal.} \end{cases}\tag{4}\end{align*}

where $\mu M_{i}$ shows various severity classification models for Chikungunya disease. $M_{7}$ shows the proposed ACGA-RF. $H_{0}$ and $H_{A}$ define the null and alternate hypotheses, respectively. ANOVA table consists of various attributes such as a sum of squares ( $SS$), degrees of freedom ( $df$), the mean sum of squares ( $MS$), F-statistics ( $F$), and P-value ( $P$). If $P$ value of $F$ is lesser than the level of significance, then we can reject $H_{0}$) and conclude that the models are significantly different from each other.

**FIGURE 3.**
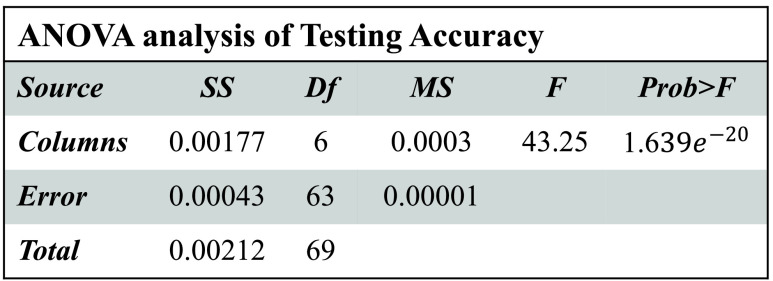
ANOVA analysis of severity classification models for Chikungunya disease in terms of testing accuracy.

**FIGURE 4.**
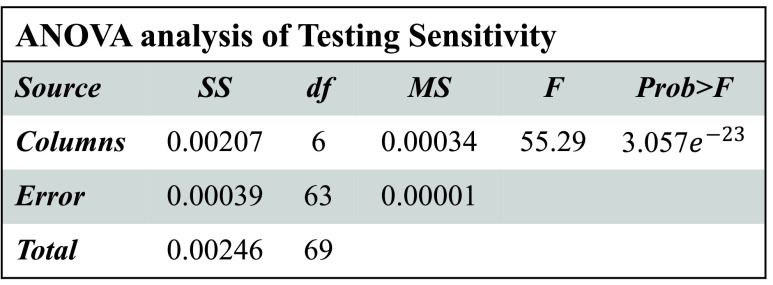
ANOVA analysis of severity classification models for Chikungunya disease in terms of testing sensitivity.

**FIGURE 5.**
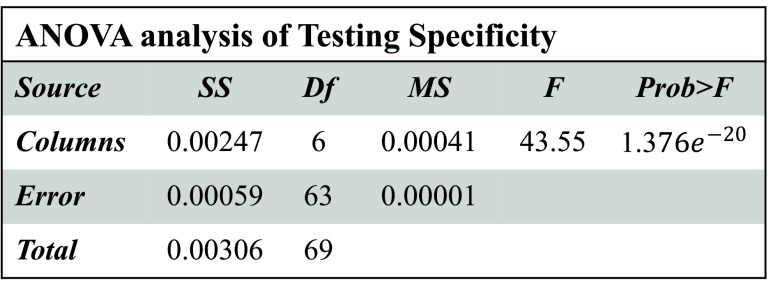
ANOVA analysis of severity classification models for Chikungunya disease in terms of testing specificity.

**FIGURE 6.**
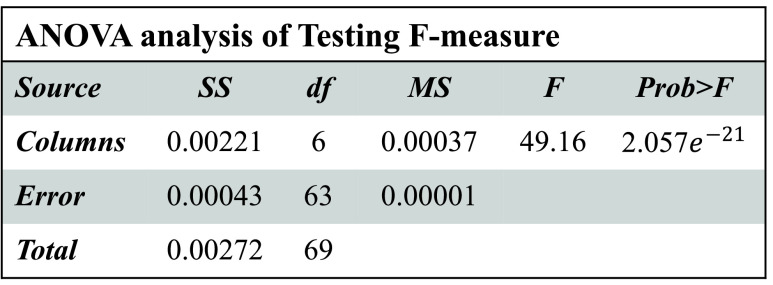
ANOVA analysis of severity classification models for Chikungunya disease in terms of testing F-measure.

**FIGURE 7.**
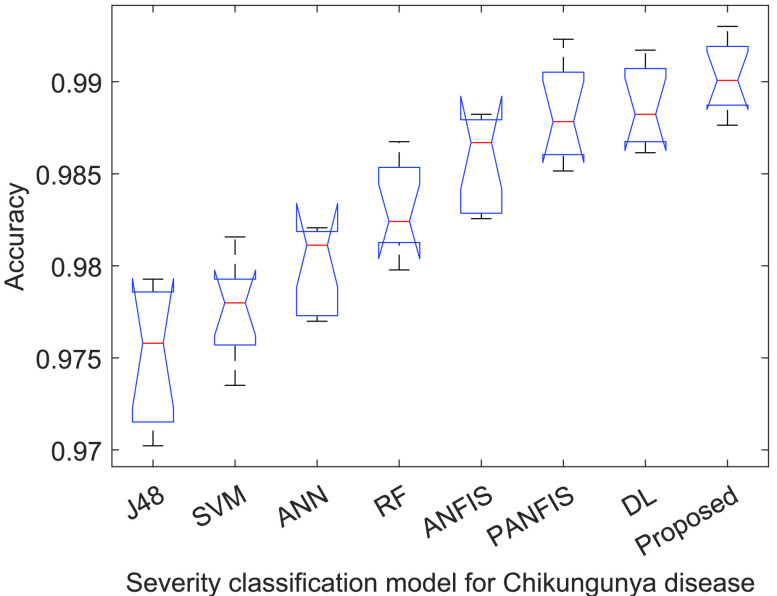
Accuracy analysis of ACGA-RF model.

**FIGURE 8.**
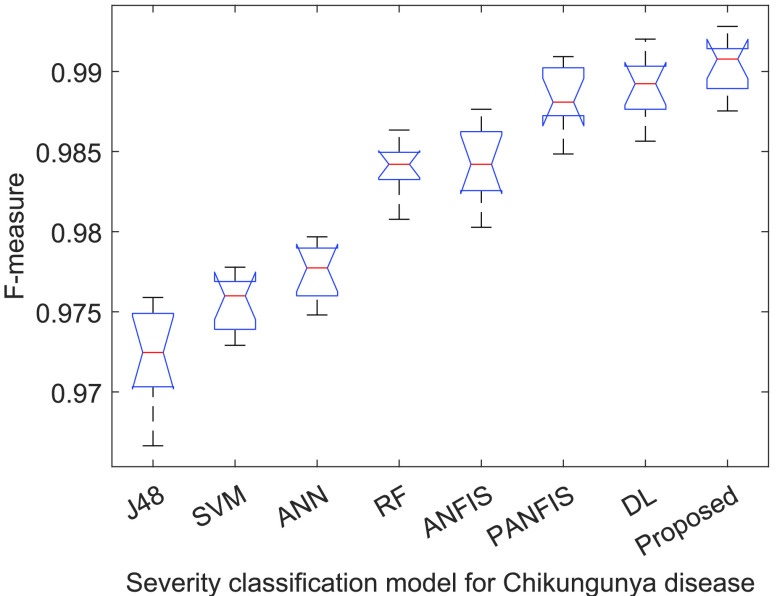
F-measure analysis of ACGA-RF model.

**FIGURE 9.**
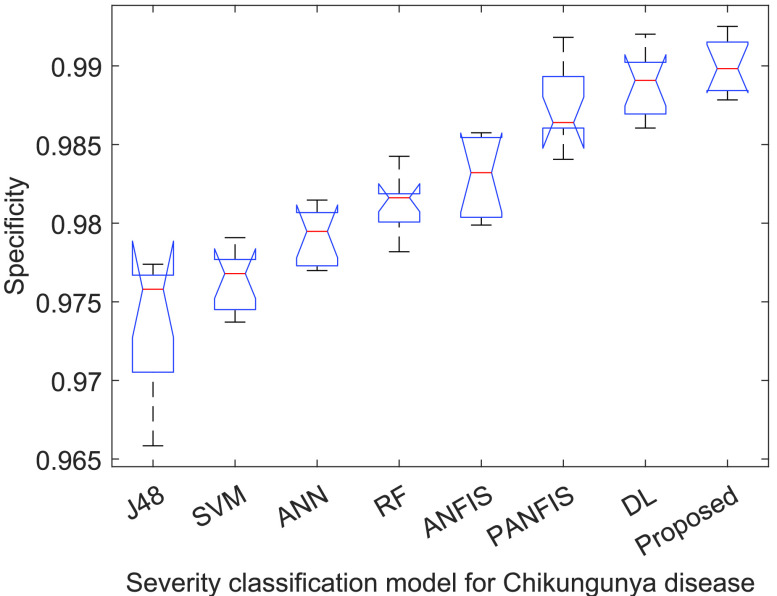
Specificity analysis of ACGA-RF model.

**FIGURE 10.**
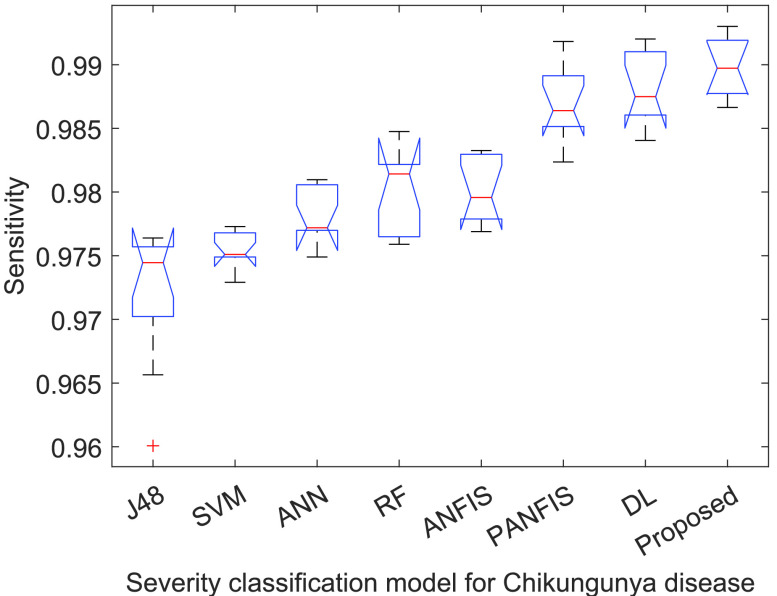
Sensitivity analysis of ACGA-RF model.

Figures 3–6 show that $H_{A}$ is accepted for all the considered performance metrics as the evaluated $p-values$ are lower than 0.01. Thus, the performance of different models is significantly different from each other. But, it is not possible to find which technique outperforms the others. Thus, the boxplots are obtained to evaluate which technique performs significantly better than the others (see Figures 7 to 10).

Figure 7 demonstrates the accuracy analysis among ACGA-RF and the existing recognition Chikungunya disease recognition models. It is clearly shown that ACGA-RF achieves remarkably significant and consistent accuracy values. Compared to the existing Chikungunya disease recognition models, ACGA-RF achieves 1.3822% improvement in terms of accuracy.

Figure 8 shows F-measure analysis among ACGA-RF and the existing Chikungunya disease recognition models. ACGA-RF outperforms the existing Chikungunya disease recognition models by an average improvement of 1.4145%.

Figure 9 shows the specificity analysis among the existing models and the proposed Chikungunya disease recognition model. ACGA-RF achieves a 99.21% (median) value, which is significantly better than the competitive models by an average improvement of 1.3972%. Therefore, ACGA-RF can efficiently diagnose normal risk Chikungunya disease-infected patients. Figure 10 shows the sensitivity analysis among the existing models and proposed Chikungunya disease recognition model. ACGA-RF achieves 99.24% (median) value, which is significantly better than the competitive models by 1.4172%. Therefore, ACGA-RF can efficiently diagnose the high risk Chikungunya disease patients.

It is found that ACGA-RF takes on an average of 1 hour 36 minutes during the training process. During the testing process, it takes only 2.746 minutes to achieve the results. Additionally, ACGA-RF provides the testing results on an average of 1.674 seconds for a single patient. Therefore, ACGA-RF can be used for real-time applications.

### D. Discussion

Table 5 shows the comparison among the proposed model with state-of-the-art machine learning and deep learning models. It is found that in [8], J48 was utilized for severity classification of Chikungunya disease. It achieved the sensitivity, specificity, F-measure, and accuracy values as 0.935, 0.965, 0.827, and 92.7865, respectively. Fuzzy-C means (FCM) [9] achieved the sensitivity, specificity, and accuracy values as 0.867, 0.888, and 0.934, respectively. PANFIS [10] achieved sensitivity, specificity, F-measure, and accuracy values as 0.9578, 0.9787, 0.9431, and 0.9871, respectively. DL based Chikungunya diagnosis model achieved sensitivity, specificity, F-measure, and accuracy values as 0.9831, 0.9763, 0.9874, and 0.9818, respectively. Compared to these models, ACGA-RF achieved a sensitivity, specificity, F-measure, and accuracy values of 0.9943, 0.9905, 0.9968, and 0.9936, respectively. Therefore, the proposed ACGA-RF achieved significantly better results than the existing models in terms of accuracy, specificity, sensitivity, and F-measure by 1.3822%, 1.3972%, 1.4172%, and 1.4145%, respectively.

**TABLE 5:** Comparative Analysis Among the Proposed ACGA-RF, DL, and State-of-the Art Models

Model	Accuracy	Sensitivity	Specificity	F-measure
J48 [8]	0.9278	0.935	0.883	0.827
FCM [9]	0.934	0.904	0.912	−
ANN [10]	0.9674	0.9438	0.9617	0.9328
PANFIS [10]	0.9871	0.9578	0.9787	0.9431
DL	0.9831	0.9763	0.9874	0.9818
Proposed ACGA-RF	0.9943	0.9905	0.9968	0.9936

## VI. Conclusion

In this paper, a cloud-based CPS is designed and implemented for the recognition of Chikungunya disease. The proposed system is divided into two main categories, i.e., physical space and cyberspace. Once, the data related to user-health are collected, it is stored in the cloud sub-system layer. An evolving RF model was proposed for the severity classification of Chikungunya disease by using ACGA. ACGA can efficiently optimize RF architecture to achieve better results with better computational speed. The comparative analysis demonstrates that ACGA-RF achieves significantly better testing performance than the existing models in terms of accuracy, specificity, sensitivity, and F-measure by 1.3822%, 1.3972%, 1.4172%, and 1.4145%, respectively. Thus, the proposed Chikungunya disease recognition model is beneficial for real-time medical applications.

In near future, the deep transfer learning models can be used to obtain more efficient results. Further novel meta-heuristic techniques can be designed to efficiently tune the deep learning architectures. Also, the proposed model can be applied to other kinds of datasets.

## Conflict of Interest

The authors declare that no conflict of interest.
